# A process evaluation of the scale up of a youth-friendly health services initiative in northern Tanzania

**DOI:** 10.1186/1758-2652-13-32

**Published:** 2010-08-23

**Authors:** Jenny Renju, Bahati Andrew, Kija Nyalali, Coleman Kishamawe, Charles Kato, John Changalucha, Angela Obasi

**Affiliations:** 1 National Institute for Medical Research, Mwanza Centre, Mwanza, Tanzania; 2 Liverpool School of Tropical Medicine, Liverpool, UK; 3 African Medical and Research Foundation, Mwanza, Tanzania

## Abstract

**Background:**

While there are a number of examples of successful small-scale, youth-friendly services interventions aimed at improving reproductive health service provision for young people, these projects are often short term and have low coverage. In order to have a significant, long-term impact, these initiatives must be implemented over a sustained period and on a large scale. We conducted a process evaluation of the 10-fold scale up of an evaluated youth-friendly services intervention in Mwanza Region, Tanzania, in order to identify key facilitating and inhibitory factors from both user and provider perspectives.

**Methods:**

The intervention was scaled up in two training rounds lasting six and 10 months. This process was evaluated through the triangulation of multiple methods: (i) a simulated patient study; (ii) focus group discussions and semi-structured interviews with health workers and trainers; (iii) training observations; and (iv) pre- and post-training questionnaires. These methods were used to compare pre- and post-intervention groups and assess differences between the two training rounds.

**Results:**

Between 2004 and 2007, local government officials trained 429 health workers. The training was well implemented and over time, trainers' confidence and ability to lead sessions improved. The district-led training significantly improved knowledge relating to HIV/AIDS and puberty (RR ranged from 1.06 to 2.0), attitudes towards condoms, confidentiality and young people's right to treatment (RR range: 1.23-1.36). Intervention health units scored higher in the family planning and condom request simulated patient scenarios, but lower in the sexually transmitted infection scenario than the control health units. The scale up faced challenges in the selection and retention of trained health workers and was limited by various contextual factors and structural constraints.

**Conclusions:**

Youth-friendly services interventions can remain well delivered, even after expansion through existing systems. The scaling-up process did affect some aspects of intervention quality, and our research supports others in emphasizing the need to train more staff (both clinical and non-clinical) per facility in order to ensure youth-friendly services delivery. Further research is needed to identify effective strategies to address structural constraints and broader social norms that hampered the scale up.

## Background

There is increasing recognition of the need to break down the barriers that prevent young people from accessing quality health care [1-5]. This is especially so for sexually transmitted infection (STI) and reproductive health services in sub-Saharan Africa, which are particularly vulnerable to many cultural and social barriers to access and uptake [6,7]. There are a number of examples of successful small-scale, youth-friendly services (YFS) interventions aimed specifically at improving services for young people in this area [3,8-12]. However, these projects are often short term and have low coverage [13]. In order to have a significant impact on young people's reproductive health, these initiatives must, in reality, be implemented over a sustained period and on a large scale [14].

Furthermore, little is known about the factors that facilitate or inhibit the effective scale up of YFS programmes, especially those aimed at improving reproductive health. Nor is there adequate information about the effect of scale up on intervention quality and implementation [10,14-16]. In small-scale, non-governmental organization (NGO) or research-led initiatives, implementers are often specially selected, highly trained and well remunerated. In contrast, scale-up programmes are usually government led and reliant on staff with lower levels of training and motivation.

The National Adolescent Friendly Clinic Initiative in South Africa is one of the few YFS programmes that has been scaled up and evaluated. It was implemented nationally by building the capacity of health workers and establishing national standards and criteria for adolescent health care in public clinics across South Africa [17]. The evaluation reported improvements in the youth friendliness of intervention clinics, specifically in terms of health workers' knowledge of adolescent rights and non-judgmental attitudes [18]. However, there was limited in-depth analysis of the implementers' and young people's point of view.

This paper presents a process evaluation of the scale up of a model YFS intervention from 18 to 177 health units. The intervention was initially designed and evaluated in 60 rural villages as part of the MEMA kwa Vijana (MkV1) community, randomized trial in Mwanza region, northwest Tanzania [19-21]. The current paper evaluates the quality of health worker training and intervention implementation within the 10-fold scale up of the YFS component of the intervention by examining key facilitating and inhibitory factors from both user and provider perspectives.

### Study setting

The study was conducted in four of Mwanza Region's eight districts, each of which has a semi-urban administration and largely rural population. Within the four districts, there are six hospitals, 24 health centres and 154 dispensaries. The cadre of health worker varies at each level. Dispensaries are run by medical assistants, accompanied by a nurse, a nurse midwife and possibly a lab technician. A health centre should be run by a clinical officer, and a district hospital run by a medical officer, supported by all other cadre of staff. However, shortages of health workers in all types of health facilities mean lower cadre staff often work above the level for which they are qualified.

### The intervention

The content and design of the original MkV1 Adolescent Sexual and Reproductive Health (ASRH) programme have been described in detail elsewhere [19-21]. However, in brief: teacher-led, peer-assisted ASRH lessons in primary school, youth-friendly services and community awareness-raising activities were implemented after in-depth training and with continued supervision from the African Medical and Research Foundation (AMREF). Although the programme showed no impact on biomedical outcomes among young people, the intervention showed substantial and sustained improvement in their knowledge, some reported attitudes and reported sexual behaviours in the medium (three years) [21] and long term (eight years) [22]. The programme also showed beneficial impact among teachers and health workers [23]. In particular, an evaluation of attendance at the health units showed that training staff to provide more youth-friendly health services increased the utilization of health services for suspected STIs by young people, especially among young men [24].

For these reasons, and in line with various Tanzanian national policies [25], a phased scale up (known as MkV2) commenced in June 2004 with the objective of extending the programme across all schools and health units in the participating districts by the end of 2008. This paper focuses on the scale up of the YFS component only.

**Figure 1:**
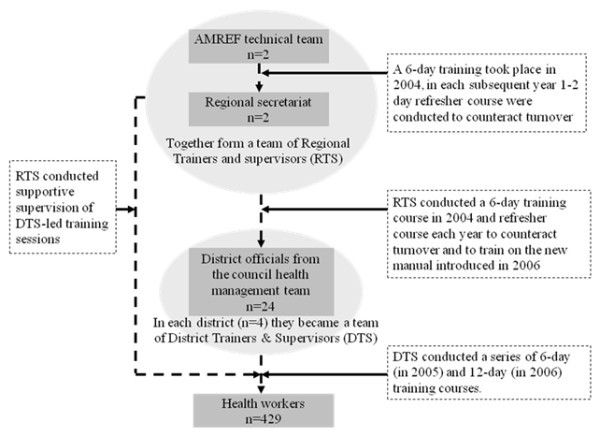
**Training cascade adopted during the scale up of the MkV health component**.

At its core, the YFS intervention relied on the in-service training of health workers at facility level. As with the rest of the MkV programme, the YFS component had been specifically designed to be scaled up through existing government structures [20]. However, the reality of implementation through a training cascade of local government officials, as opposed to training by a dedicated NGO team, meant that several modifications were made to both the content and implementation of the training given. In particular:

i. During MkV1, all aspects of intervention implementation were closely supervised by AMREF and the London School of Hygiene and Tropical Medicine, and all health workers were directly trained by AMREF staff. By contrast, health workers in the scale up (MkV2) were trained exclusively by local government officials, who had themselves been trained in cascade fashion (Figure 1).

ii. In MkV1, health workers received an annual refresher course after their initial training, whereas in MkV2, health workers were trained once only.

iii. In MkV1, the AMREF team set the health worker selection criteria, whereas in MkV2, selection was at the discretion of the district authorities.

Finally, towards the end of 2005, the Ministry of Health and Social Welfare (MoHSW) launched a YFS training manual (Manual 2) as part of a new Adolescent Health and Development Strategy [26]. This drew on MkV1 and MkV2 experiences, and senior MkV2 implementers had key advisory roles within its development and implementation. The new government manual differed from the MkV training (Manual 1) in several key respects, most notably in duration and contents (Table 1). Health worker training was therefore conducted in two rounds (Round 1: January-June 2005; Round 2: October-July 2007), so that the programme could follow the revised MoHSW guidance and use the new MoHSW training manual for Round 2.

**Table 1:** Comparison between the two manuals used when scaling up the MkV intervention

	Manual 1 MEMA kwa Vijana developed manual	Manual 2 Ministry of Health and SocialWelfare-developed manual
**Duration**		
*Number of days*	6 days	12 days

**Delivery**		
*Time per day*	9 am-4 pm	9 am-6 pm

*Intensity*	Time for breaks and reflection of content	A lot of information intended for each day, making it difficult to complete all the tasks as planned

*Teaching strategy*	Each topic began with participatory brainstorming prior to new material	New material introduced straight away, spot checks were used at the end of each activity

*Ongoing evaluation*	None: evaluation at end of training	Daily evaluation with answers provided

**Intervention materials**		
*Materials*	Training manual only	Trainers' manual & participant's handout

*Language*	English & Swahili versions	English only

**Content analysis^a^**		
*Refs and details*	No references	For each chapter, there are references and statistics

*Confidentiality*	1	3

*Rape*	1	3

*Menstrual cycle*	1	2

*Condoms*	1	3

*Gender*	1	2

*Contraception*	2 (except condoms)	1

*HIV*	3	1

*STIs*	3	1

*Stages of Adolescence*	3	1

*Counselling*	3	1

^a^Key: 1 = Stand alone topic 2 = Not covered 3 = Covered within another topic/less detail

## Methods

### Design

The quality and immediate outcomes of the YFS training were evaluated using pre-and post-training questionnaires, training observations and informal interviews among health workers during training. Intervention implementation was evaluated by baseline and follow-up qualitative surveys in a small number of health facilities to examine provider perspectives, and by a simulated patient study to examine user perspectives. The simulated patient study included linked health worker interviews to further explore issues arising in the patient-client interaction (Table 2).

**Table 2:** Timeline of implementation and evaluation activities involved in the MkV health component between 2004 and 2007

Date	Implementation activity	Evaluation activity
***Oct-Nov 2004***	**Training of 24 DTs**	
	6-day training	
***Jan-June 2005***	**Training with Manual 1:**	**Training evaluation**
	Eight training sessions of 208 health workers (6-day training)	- 16 days observation of five training sessions
		- 208 pre- & 203 post-training questionnaires
		- Informal interviews
***Feb-March 2005***		**Baseline study^†^**:
		Interviews with 20 HWs prior to receiving the training in 2006
***April 2006***	**Introduction of Manual 2:**	
	12-day training of 24 DTs with MoHSW manual	
***June 2006***		**Simulated patient study:**
		One SP visits to 15 health units: eight with trained HWs (intervention) & seven without trained HWs (control)
***August 2006***		**Health worker interviews:**
		Follow-up interviews with 30 HWs from the same health units visited by the SP
***Oct 2006 to July 2007***	**Training with Manual 2:**	**Training evaluation**
	Eight training sessions of 221 HWs (12-day training)	- 20 days observation of three - training sessions
		- 221 pre- & post-training questionnaires
***August 2007***		**Follow-up study:**
		Interviews with 15 MkV-trained HWs, two group discussions & two interviews with non-MkV-trained HWs

Key:

**^† ^**The intervention had already begun prior to the research team being in place. Therefore ongoing training were evaluated prior to a baseline study being conducted in geographically separate areas.

**Acronyms: **DTs - district trainers; HWs - health workers; MkV - MEMA kwa Vijana (a multi-component adolescent health project); MoHSW - Ministry of Health and Social Welfare

### Training evaluation

Carefully piloted self-complete questionnaires were administered immediately before and after their training. The questionnaire took around 30 minutes to complete, and consisted of multiple choice questions to assess knowledge on STI/HIV/AIDS transmission and prevention, knowledge on pubertal changes, and attitudes towards condoms, confidentiality and young people's rights to treatment, as well as to collect socio-demographic information.

Trained researchers conducted observations of training sessions in order to document the coverage, attendance, selection, motivation, experiences, attitudes, perceptions, characteristics, ownership, training content and delivery, levels of support, logistics and other external factors. The researchers used pre-defined checklists to guide the observations and wrote detailed reports for each session they observed. Researchers also used the times before, between and after classes for *ad hoc *informal interviews with the participants and facilitators in order to clarify any points observed throughout the sessions, as well as to build rapport. Regular supervision was conducted to ensure consistency in the data collection and documentation.

### Facility-level implementation evaluation

The qualitative baseline and follow-up surveys were conducted in a sample of eight health units. All wards in each district were stratified as either rural or peri-urban. Health centres were stratified by hospitals, health centres and dispensaries. In each district, two health units were selected; the sample included two hospitals, one health centre and five dispensaries.

Semi-structured interviews and group discussions were conducted at baseline (three months prior to training) and 6.5 to 10 months after training among all health workers in selected health units. The interviews and group discussions addressed health workers' views of young people and knowledge on, attitudes towards, and experience of different aspects of YFS. In addition, data was collected on contextual and environmental factors, including: staffing; the space, layout and condition of the rooms; and the availability of equipment, drugs, information materials and condoms and the functioning of the management information systems. Similar guides were used in both the baseline and follow-up surveys to enable comparison.

A simulated patient (SP) or "mystery client" study was conducted to assess the effect of the intervention on the quality of delivery of YFS. The timeline for intervention implementation was devised by the districts and was beyond the control of the research team. A purposive sampling strategy was therefore adopted to ensure representation from each district and from each type of health unit. Two pre-intervention (control) and two post-intervention health units were selected per district, and the sample included nine dispensaries, three health centres and four hospitals.

Three scenarios and checklists (Table 3) were developed (condom request, family planning request and STI query) in consultation with the AMREF implementation team and clinical officers. These were based on the experiences of other SP studies conducted in the same area [27] and on the MoHSW adolescent health strategy standards for YFS [26].

**Table 3:** Summary of simulated patient scenarios and frequency of each scenario

Scenario	Details
**STI query scenario**	• The young person (YP) is worried that s/he has an STI after having had sex for the first time with a new partner whom s/he has since heard had an STI.
	• YP does not yet have any signs or symptoms.
	• The YP is also worried his/her parents will find out.

**Condom request scenario**	• YP requests condoms because he is sexually active, has heard about STDs/HIV, and has heard that condoms may prevent them.
	• YP has also heard that condoms are free at health units.
	• YP is worried that they contain HIV or that they have holes in them.

**Family planning request scenario**	• A 16-year-old schoolgirl had sex for the first time one month ago with a boyfriend of 2 months who is also a pupil.
	• She is not using contraception and is worried about getting pregnant.
	• She knows very little about contraceptives, wants to avoid pregnancy, and is afraid to talk her parents

The recruitment and training of the young people who would act as SPs took place in five stages (sensitization, selection, consent, training and pilot). The six-day intensive training included how to operate and hide tape recorders, and culminated in a pilot in eight health units in Mwanza town. Thereafter, four (selected from a shortlist of eight) SPs, blinded to the intervention status of the health facilities, conducted the final SP scenarios in the clinics. Detailed checklists were used during debriefs, which took place immediately after each SP reached the project vehicles.

Two months after the SP study, a final round of semi-structured interviews was carried out among health workers from the health units that had been visited by the SPs. Intervention-trained health workers were not preferentially selected. Instead, a sample of health workers from either the outpatient department or the maternal child health department was interviewed. This was done in order to include any health worker that could have had a consultation with the SPs a few months earlier. Interviews were structured around topic guides similar to those used in the previous interview and/or group discussion in order to aid comparison across the sites.

### Analysis

Questionnaire data were double entered, verified and cleaned using Dbase IV (Borland International, Scotts Valley, California). Univariate analysis was conducted using STATA Version 8 (STATA Corporation, College Station, Texas, USA). The interview and group discussion transcripts were anonymized and coded in QSR NVIVO version 2.0 (Rouge Wave Software Inc., Yves Roumazeilles) and analyzed using a thematic content approach. The recordings from the SP consultations and the SP debriefs were transcribed. Researchers blinded to the intervention status of the SP consultations scored the five criteria according to a pre-set scoring scheme (Table 4). The average for each criterion were calculated by intervention or control group and expressed as a percentage of the maximum possible score.

**Table 4:** Knowledge and attitudes of health workers before and after the youth friendly services training, between 2005 and 2006.

	2005				2006				***2006 v 2005***^***†***^
	
*% of HWs with all correct/desired answers*	Pre n = 208	Post n = 203	RR (95% CI)		Pre n = 221	Post n = 225	**RR (95 **% **CI)**		*P value*
*Knowledge on*									
*HIV/AIDS*	78.8	87.1	1.11*	(1.00, 1.21)	91.8	99.6	1.08**	(1.04, 1.13)	*p = 0.32*
*puberty*	83.3	93.6	1.12**	(1.05, 1.22)	81.2	85.9	1.06*	(1.02, 1.16)	*p = 0.19*
*STDs*	34.5	29.8	0.86	(0.63, 1.18)	28.9	57.7	2.00**	(1.54, 2.59)	*p < 0.001*
*Attitude relating to*									
*stigma*	89.0	94	1.06	(0.98, 1.12)	80.5	93.1	1.16**	(1.07, 1.26)	*p < 0.001*
*young people*	77.1	97.3	1.26**	(1.15, 1.38)	69.9	92.2	1.32**	(1.19, 1.47)	*p = 0.02*
*condoms*	96.4	97	1.01	(0.97, 1.04)	96.7	97.3	1.01	(0.97, 1.04)	*p = 1*
*Condoms use amongst school pupils*	83.3	97.5	1.17	(0.98, 1.06)	90.7	96.4	1.06*	(1.01, 1.12)	*p < 0.001*
*Confidentiality*	66.7	82.2	1.23**	(1.09, 1.41)	67.6	92.0	1.36**	(1.22, 1.52)	*p < 0.001*
*Young people's right to treatment*	56.4	74.9	1.33**	(1.17, 1.60)	54.6	74.5	1.36**	(1.17, 1.60)	*p = 0.21*

*significant at 0.05 level

**significant at <0.01 level

^†^Adjusted for baseline differences in education, previous training, HIV knowledge and attitudes relating to young people

### Ethics

The Tanzanian Medical Research Coordinating Committee approved this study. Approval for the study was also obtained from the offices of the Regional and District Commissioners, the Regional Medical Officer and the District Medical Officers. The head of each health unit, the parents of the simulated patients, the simulated patients and health workers individually consented to take part in the study.

## Results

### Evaluation of the training

#### Coverage

A total of 429 health workers from 177 health units were trained by the district trainers in 16 training session Of these, 208 were trained in Round 1 using Manual 1 (seven sessions), and 221 in Round 2 using Manual 2 (nine sessions). Questionnaires were available from 208 (100%) (pre) and 203 (98%) (post) health workers trained in Round 1, and from 221 (100%) (pre) and 221 (100%) (post) health workers trained in Round 2.

The number of health workers selected for training varied by type of health unit, (one to two per dispensary, two to four per health centre, and four to six per hospital,) and represented, on average, 50% of clinical staff. Respondents therefore came from a range of cadres: clinical officers (26%), assistant clinical officers (17%), medical attendants (25%), nurse midwives (12%), public health nurses (9%) and others (medical officers, assistant medical offices, laboratory technicians, pharmacists). The majority of respondents were from dispensaries (69%).

There were no significant differences between the demographic characteristics of health workers participating in either of the two training rounds. The distribution was as follows: gender (57% female), religion (53% Catholic), tribe (51% Sukuma, the predominant ethnic group), education level (21% had completed primary school and a further 40% had completed four years of secondary school), and previous training (47% had previous training in STIs and only 27% in HIV/AIDS).

#### Training implementation

Thirty-four person days of observations were conducted covering all or part of eight of the 16 training courses. Observations found district-led training to be well conducted, with minimal support from the regional trainers. District trainers' confidence and ability in conducting the training (particularly using participatory techniques) improved over time. However, there were some areas of difficulty: (i) lack of health worker knowledge (confirmed by pre- and post-training questionnaires) hampered teaching about the menstrual cycle; (ii) insufficient time (one session only) hampered teaching about counselling; and (iii) mixed gender and cadre training led to variable participation, with higher cadre male health workers dominating sessions.

The notable differences between the two rounds were initial logistical problems and funding delays in Round 1. However, these reduced when AMREF provided cars to the districts and with increasing district trainers' capacity to plan and request funds. Also, in the second round, the facilitators faced challenges working with Manual 2. The manual was in English, yet Swahili was used in sessions. Real-time translation led to confusion, and researchers observed some variations in the messages that the facilitators were relaying.

#### Impact of training on health worker knowledge and attitudes

Questionnaires from 429 health workers confirmed that the district-led training significantly increased HIV, AIDS, STI and puberty knowledge (RR ranged from 1.06 to 2.0) and improved their attitudes towards condoms, confidentiality and young people's right to treatment (RR range: 1.23-1.36) across both years (Table 4). After adjustment for education and previous training, Round 2 produced significantly greater changes in knowledge of STIs (p < 0.001) and attitudes towards stigma (p < 0.001), young people (p = 0.02), condom use among school pupils (p < 0.001) and confidentiality (p < 0.001) than Round 1.

### Evaluation of the implementation of youth-friendly services at facility level

For the qualitative surveys, semi-structured interviews were conducted among 20 health workers at baseline and 15 intervention-trained health workers and two non-intervention-trained health workers, and two group discussions with non-intervention trained health workers at follow up.

#### Health workers' attitudes and experience of reproductive health services at baseline

Twenty health workers (50% male) were interviewed at baseline. Nine were from dispensaries, eight from hospitals and three from health centres. All the health workers had had some previous training in HIV and STIs, although none had been previously trained in YFS.

Health workers stated that generally more women and very few young people attended services. They believed that this was because many young people were shy and were more likely to self-treat, often culminating in the late presentation at the health facility:

Those that come are already very advanced, for example if he is male sometimes you can see that his penis is already weeping, it is normally very bad. [Interview with Assistant Clinical Officer]

Health workers also felt that their different employment arrangements hampered service delivery. Some were central government employees and complained of delayed salaries; others were district council employees and complained of insufficient salaries; and mission employees (from one mission hospital) complained about long working hours without overtime pay. These factors, compounded by the staff shortages, demotivated the health workers.

Health workers from 50% of the sample health units reported that room shortages caused longer waiting times and compromised privacy levels. The observations of the health facilities at baseline confirmed that all but one health unit had a shortage of rooms. More than half had no clear 'patient' flow from the reception to the consultation room, meaning that a young person would have to pass through the waiting area a number of times during his or her visit.

Only one of the 20 health units had adequate structures in place to ensure privacy and confidentiality. Generally, rooms did not have doors and/or complete walls, and consequently, private consultations could be overheard. All health facilities had adequate equipment and furniture to provide services, and none reported problems with drug procurement or reporting mechanisms. In all but one facility, condoms were available; however, they were often only accessible from the health workers' room.

The baseline study concluded that improvements were needed in order to facilitate the provision of YFS, in terms of motivating and training health workers and addressing some infrastructural constraints.

#### Health worker attitudes and experience of reproductive health services at follow up

Fifteen intervention-trained health workers (48% male) were interviewed at follow up. Eight were from dispensaries, six from hospitals and one from a health centre. Of these, six (40%) had been trained using Manual 1. Three group discussions and two interviews were conducted with non-intervention-trained health workers.

While intervention-trained health workers appreciated the value of the training and reported that they were happy with the selection criteria, those who had not received training disagreed. Non-intervention-trained health workers complained that the selection favoured certain people, for example, more senior health officials, whom, they stated generally spend less time with young people:

For example, the matron went on the training but she is not seen most of the time as she is in the office and therefore not able to help young people. (Respondent 1)

Why was it that all those that went on the training were all the senior people? (Respondent 2)

It is true that senior people were chosen; these people do not understand young people. (Respondent 3)

[Focus group discussion with non-intervention-trained health workers]

The semi-structured interviews supported the findings of the training evaluations. Trained health workers illustrated an increased recognition of young people's need for information and advice. They reported themselves to be more aware of the importance of confidentiality, privacy and respect for young people:

Before I would tell people what I discussed with young people; however I realize that this led them to not open up to me. [Intervention-trained health worker]

Shortage of staff, time and resources challenged some of the health workers in the provision of YFS. Further, many of the same structural constraints noted during the baseline study, for example, shortage of rooms, remained a challenge to health workers during the follow-up study:

Our building has no space for privacy, even a little as we have no special room that is private in order to give out private information. [Intervention-trained health worker]

#### Impact on the perceived "friendliness" of health service delivery to young people

Fourteen of the intended 16 sites were actually included in the SP study. Two pre-intervention sites were not included. In the first (a hospital), the SP failed to record the consultation, and in the second (a dispensary), normal services were disrupted by an immunization campaign. Data from the 14 SP visits showed that, overall, health workers performed better in intervention health facilities for two of the three scenarios (family planning query and condom request) (Table 5).

**Table 5:** Scoring scheme and scores achieved by each health facility based on the simulated patient visits.

	STI scenario		Family planning scenario		Condom request	
	Cont^‡^	Int^†^	Cont^‡^	Int^†^	Cont^‡^	Int^†^
Number of SP visits	2	3	2	3	3	2

*Area (possible score per SP)*	*Average score (% of total possible score)*					
Welcome (4)^a^	2.0 (50%)	0.7 (18%)	1.5 (38%)	1.7 (43%)	1.7 (43%)	3.0 (75%)
Information (2) ^b^	0.5 (25%)	0.0 (0%)	0.0 (0%)	1.0 (50%)	0.7 (35%)	0.5 (25%)
Counseling (4) ^c^	3.0 (75%)	0.7 (18%)	2.0 (50%)	3.3 (83%)	1.3 (33%)	4.0 (100%)
General attitudes(10) ^d^	6.5 (65%)	2.3 (23%)	5.0 (50%)	6.7 (67%)	3.3 (33%)	5.5 (55%)
Privacy (6) ^e^	3.0 (50%)	2.7 (45%)	3.0 (50%)	6.0 (100%)	0.0 (0%)	6.0 (100%)

**Total (31)**						

Key of score criteria

^‡ ^Cont = Control health units - no YFS trained health workers

^† ^Int = intervention health unit - with YFS trained health workers

**^a ^Welcome: **Two points for each of the following, maximum **four points**: 1. SP was greeted in a friendly manner and 2. SP waited for a short time or was spoken to earlier to inform him/her on the process.

**^b ^Information: **One point for each of the following maximum **two points**: 1. SP received the information they needed and 2. The HW conducted a condom demonstration.

**^c ^Counselling: **Two points for each of the following maximum **four points**: 1. SP received reassurance and advice about all their concerns, 2. HW listened to the SP as they recounted the scenario and in the subsequent discussions.

**^d ^General attitudes: **Different points for each of the following maximum **ten points**: 1. HW was non-judgmental (4), 2. the SP was given enough time to talk (3) 3. The SP felt they could ask questions (3)

**^e ^Privacy: **Two points for each of the following maximum **six points**: 1. no-one else was in ear shot, 2. SPs were treated in a private room, 3. no-one else was in the room

Health workers' general attitudes to young people and understanding and respect for privacy were scored higher than the other assessment criteria (welcome, information and counselling). Waiting times were generally shorter in the intervention health units. In five intervention health units SPs were prioritized over other older patients. In five control health units, SPs had to wait up to two hours; no SP was prioritized.

Fewer SPs in intervention health units were requested to pay for services. However, in many health units (both intervention and control) consultations were rushed, did not collect comprehensive patient or sexual histories, and did not provide adequate information, leaving many of the SP's questions unanswered:

- **Clinician: **What is your name?

- **Clinician: **Where do you live?

- **Clinician: **How old are you?

- **Clinician" **What is your problem?

- **SP: **[SP recounted the condom scenario (see Table 3)]

- **Clinician: **Do you feel pain at all when you urinate?

- **SP: **No

- **Clinician: **You must come with 1500/= so that you can be tested

- END OF CONSULTATION -

[SP recording from a control health facility]

There was no privacy because the doctor spoke with a loud voice and people who were near were listening, there was no door, there was only a curtain! [SP report during debrief from a control health unit]

Further, in both pre- and post-intervention health units, SPs were consistently asked to recount their scenarios to the receptionist in view and earshot of others. Finally, intervention health units performed poorly in the STI scenario, scoring lower than control health units in all five criteria.

Two months after the SP study, 30 interviews took place with health workers from the same facilities (15 intervention, 15 control). The sample included health workers of different cadres as follows: clinical officers (9), assistant clinical officers (3), senior nurses (3), mother and child health attendants (11), medical attendants (3), and a public health nurse (1). On the day of the research, all those selected were working in departments (maternal child health and outpatients), which the SPs visited two months earlier. Only 40% (6) of health workers from the intervention health units had actually been trained by MkV; four intervention-trained health workers had travelled, two had been transferred, and in three health units, either none or only one health worker had been trained.

The interviews suggested that, overall, intervention-trained health workers displayed higher levels of knowledge and a better understanding of the needs of young people than those with no intervention training. Intervention-trained health workers responded better to both the hypothetical STI and family planning scenarios; they were more aware that family planning services were also suitable for young people and not just married women or women with children (as was mentioned by non-intervention-trained health workers):

At first I thought that family planning methods were for older women ... but after the training in youth-friendly services it was clear that they are even for young people, we are supposed to provide young people with these services if they need them. [Intervention-trained health worker]

However, there was little difference between the non-intervention-trained health workers from the intervention health units and the health workers from pre-intervention (control) health units.

Both trained and untrained health workers felt that young people's shyness prevented young people from coming to the health unit and, if they reach a health unit, inhibited explanation of their problems. Intervention-trained health workers reported that despite their efforts, many young people refuse to use condoms because they want to conceive:

Young people, starting as young as 10 to 16 years, refuse to wear condoms, they say that they don't want to use family planning, believing they are ready to have children or maybe they are already pregnant, then they just refuse to use condoms. [Intervention-trained health worker]

Health workers also believed that young people's poor perception of services, lack of knowledge and lack of life skills has culminated in them having poor health-seeking behaviour, preferring to self-treat or visit traditional healers, subsequently delaying their visits to the health units.

## Discussion

The studies presented here have examined the quality of training of health workers and of facility-level implementation during the scale up of an YFS intervention in four districts in Mwanza Region, Tanzania. Our study confirms that a training cascade in which district-level officials provide in-service training for facility level staff can achieve high (99%) coverage of health facilities. In addition, our data suggest that the training process was well conducted, and significantly improved health workers' knowledge and attitudes in key areas for YFS [2,4,21].

Finally, our data do suggest that the training improved the youth friendliness of some aspects of service provision at facility level. However, despite these positive findings, our study suggests that the effect of the overall intervention was limited by the small number and high turnover of trained health workers at each facility and by several other infrastructural factors explored in the research.

The study is strengthened by its use of both quantitative and qualitative methods, and the inclusion of data from all clinical cadres involved in YFS provision. The SP study was able to capture the realities of young people's experience of YFS provision. The prospective collection of data and repeat observations both pre and post intervention adds further validity to the study. Finally, the high coverage of the training in terms of questionnaires and observations and the congruence of the triangulation of the results lends weight to the outcomes.

However, there were various limitations in study design that should be noted when interpreting the results. First, the implementation timetable was out of the control of the research team and no baseline or random allocation of health units to either intervention or control arms could take place. Further, the quasi-experimental design overall means we can not exclude that the findings have been subject to confounders owing to changes in context and environment over time. In addition, the small size of the SP and baseline and follow-up qualitative studies means that we cannot exclude the possibility that the findings were due to chance. Notwithstanding these limitations, the study highlights important issues in the provision of large-scale, youth-friendly service programmes in rural communities in Africa and for the evaluation of these services overall.

Various adaptations need to be made to programmes when they are scaled up, which could potentially compromise quality [28,29]. In this case, providing one training session per health worker increased vulnerability to turnover. Health workers from one-third of all sampled health facilities had moved out of the area six to 12 months after the initial training. The district-devised training selection criteria favoured those of higher cadre, and subsequently, more people in administrative and managerial roles, rather than direct interaction with young people. The programme trained health professionals exclusively. The experiences of the SPs in our study strongly support other studies [4,14] in highlighting the need to train auxiliary staff, in particular receptionists, in order to improve the respect shown to young people and decrease waiting times and lack of privacy [30].

The content and duration of the health worker training differed between the two rounds. Our findings suggested that while both versions of the training improved health workers' knowledge and attitudes, this effect was greatest with the MoHSW manual. This is unlikely to be due to health worker differences at baseline, and more likely to be due to the differences in the training manuals (longer duration, more detailed participant materials and stronger focus on key technical areas, e.g., STIs and HIV prevention, counselling and stages of adolescence in Manual 2). However, this may be critically confounded by the fact that the trainers became more confident and competent in the second year. This programme is also a welcome example of effectively getting research into policy and practice, in that MkV1 and MkV2 contributed to the development of a national training manual, which was in some respects more effective than the interventions from which it was derived.

Of note, levels of HIV knowledge among health workers were much greater than STI knowledge at baseline and follow up. Baseline differences could be due to the priority that is given to HIV national policies, campaigns or programmes. However, follow-up levels of STI knowledge and the findings from the three STI scenario SP visits suggest that additional STI training is needed [31].

Only 40% of health workers in the intervention health units visited by SP had been trained. The interview responses suggested little difference between the untrained health workers in the intervention health units and those from control health units. This lack of difference suggests that there was little transfer of knowledge between the intervention-trained health workers and their colleagues. In line with other studies, this highlights the importance of training more or all health workers per facility [13]. This, together with the high staff turnover, also suggests that conducting refresher training more frequently would further enhance the impact [10,13,18,27]. Pre-service training would also critically increase coverage of facility staff and may be much more cost effective.

The question of the impact of scale up on intervention effectiveness can only really be answered by direct comparison to the original pilot intervention. Indeed, some similarities with the MkV1 findings were noted: specifically, improved knowledge and attitudes of health workers and some evidence of improved service delivery [24]. Although the study designs differed, the order of the improvements noted during the scale up appeared diluted. The findings from MkV1 suggested greater training improvements and more notable differences between intervention and control health units during the SP study [21,24,27]. However, true comparisons are prevented by the confounding effect of changes in environment and context that are likely to have occurred between the implementation of the pilot (1999-2002) and its eventual scale up (2004-2008).

Our research was also able to document unforeseen policy impacts of the scale-up process. In particular, MkV2 appeared to contribute to the development of the MoHSW's Adolescent Health and Development Strategy through the participation of key technical staff. Further, by adopting the manual in Round 2, it is likely that the scale up substantially improved dissemination and uptake of the new YFS policy. Finally, the improvements observed in the quality of training implemented by the district teams suggest that the scale up is likely to have built regional and district capacity, which may have benefits beyond the scope of programme.

## Conclusions

YFS training can remain well delivered and improve youth friendliness even after large-scale expansion through existing systems. Our research suggests that the scale-up process may have diluted some aspects of the intervention quality, and supports others in emphasizing the need to train more staff (clinical and non-clinical) per facility. However, intervention quality continues to be hampered by contextual factors, such as staff turnover and conditions of employment, which must be addressed if interventions are to achieve their full potential.

## Competing interests

The authors declare that they have no competing interests.

## Authors' contributions

JR was the research coordinator for the scale up of MEMA kwa Vijana (MkV2), supervised the data collection, and performed the final analysis and write up of all the components of this study and manuscript. BA was lead researcher for the data collection of all components of this study, was involved in the design, led the implementation of the simulated patients study, and co-wrote the first iteration of the manuscript. KN was involved in the design and data collection, conducted most of the training observations and analysis, and co-wrote the first iteration of training evaluation section of this manuscript. ColK performed the final statistical analyses for the health worker training data. CK led the implementation of the health component, supported the design and implementation of the research, and commented on various drafts of this paper. JC was co-principal investigator of the MkV2, and supported the research on all phases of MkV2. AO was co-principal investigator of the MkV2, oversaw the design, implementation and interpretation of all the studies in this paper, and contributed substantially to various drafts of the manuscript.

## References

[B1] Faxelid E Ahlberg BM Ndulo J Krantz I Health-seeking behaviour of patients with sexually transmitted diseases in Zambia East Afr Med J 1998 13 4 232 236 9745841

[B2] Kipp W Chacko S Laing L Kabagambe G Adolescent reproductive health in Uganda: issues related to access and quality of care Int J Adolesc Med Health 2007 13 4 383 393 18348414 10.1515/ijamh.2007.19.4.383

[B3] Tylee A Haller DM Graham T Churchill R Sanci LA Youth-friendly primary-care services: how are we doing and what more needs to be done? Lancet 2007 13 9572 1565 1573 10.1016/S0140-6736(07)60371-7 17482988

[B4] Warenius LU Faxelid EA Chishimba PN Musandu JO Ong'any AA Nissen EB Nurse-midwives' attitudes towards adolescent sexual and reproductive health needs in Kenya and Zambia Reprod Health Matters 2006 13 27 119 128 10.1016/S0968-8080(06)27242-2 16713886

[B5] Zachariah R Nkhoma W Harries AD Chantulo A Spielmann MP Mbereko MP Buhendwa L Health seeking and sexual behaviour in patients with sexually transmitted infections: the importance of traditional healers in Thyolo, Malawi Sex Transm Infect 2002 13 2 127 129 10.1136/sti.78.2.127 12081174 PMC1744435

[B6] Gregson S Adamson S Papaya S Mundondo J Nyamukapa CA Mason PR Garnett GP Chandiwana SK Foster G Anderson RM Impact and process evaluation of integrated community and clinic-based HIV-1 control: a cluster-randomised trial in eastern Zimbabwe PLoS Med 2007 13 3 e102 10.1371/journal.pmed.0040102 17388666 PMC1831737

[B7] Stephenson R Community influences on young people's sexual behavior in 3 African countries Am J Public Health 2009 13 1 102 109 10.2105/AJPH.2007.126904 19008517 PMC2636596

[B8] Sanci LA Coffey CM Veit FC Carr-Gregg M Patton GC Day N Bowes G Evaluation of the effectiveness of an educational intervention for general practitioners in adolescent health care: randomised controlled trial BMJ 2000 13 7229 224 230 10.1136/bmj.320.7229.224 10642233 PMC27271

[B9] Save the Children "Youth Friendly" pharmacies in Bolivia 2004 Save the Children: Westport

[B10] Senderowitz J Hainsworth G Solter C A Rapid Assessment of Youth Friendly Reproductive Health Services Technical Guidance Series 2003 Pathfinder International: Waterstown, MA

[B11] Shafer MA Tebb KP Pantell RH Wibbelsman CJ Neuhaus JM Tipton AC Kunin SB Ko TH Schweppe DM Bergman DA Effect of a clinical practice improvement intervention on Chlamydial screening among adolescent girls JAMA 2002 13 22 2846 2852 10.1001/jama.288.22.2846 12472326

[B12] Walker Z Townsend J Oakley L Donovan C Smith H Hurst Z Bell J Marshall S Health promotion for adolescents in primary care: randomised controlled trial BMJ 2002 13 7363 524 10.1136/bmj.325.7363.524 12217993 PMC121334

[B13] Mathews C Guttmacher SJ Flisher AJ Mtshizana YY Nelson T McCarthy J Daries V The quality of HIV testing services for adolescents in Cape Town, South Africa: do adolescent-friendly services make a difference? J Adolesc Health 2009 13 2 188 190 10.1016/j.jadohealth.2008.05.009 19167669

[B14] Smith J Colvin C Getting to scale in young adult reproductive health programs Focus on Young Adults 2000

[B15] DeJong J A question of scale? The challenge of increasing the scale of non-governmental organisations, HIV/AIDS efforts in developing countries Horizons/Alliance Project on Scaling Up HIV/AIDS Programmes 2001 Population Council: Washington, DC

[B16] Oliveira-Cruz V Hanson K Mills A Approaches to overcoming constraints to effective health service delivery: A review of the evidence Journal of International Development 2003 13 41 65 10.1002/jid.965

[B17] Dickson-Tetteh K Pettifor A Moleko W Working with public sector clinics to provide adolescent-friendly services in South Africa Reprod Health Matters 2001 13 17 160 169 10.1016/S0968-8080(01)90020-5 11468833

[B18] Dickson KE Ashton J Smith JM Does setting adolescent-friendly standards improve the quality of care in clinics? Evidence from South Africa Int J Qual Health Care 2007 13 2 80 89 10.1093/intqhc/mzl070 17277012

[B19] Hayes RJ Changalucha J Ross DA Gavyole A Todd J Obasi AI Plummer ML Wight D Mabey DC Grosskurth H The MEMA kwa Vijana project: design of a community randomised trial of an innovative adolescent sexual health intervention in rural Tanzania Contemp Clin Trials 2005 13 4 430 442 10.1016/j.cct.2005.04.006 15951245

[B20] Obasi AI Cleophas B Ross DA Chima KL Mmassy G Gavyole A Plummer ML Makokha M Mujaya B Todd J Wight D Grosskurth H Mabey DC Hayes RJ Rationale and design of the MEMA kwa Vijana adolescent sexual and reproductive health intervention in Mwanza Region, Tanzania AIDS Care 2006 13 4 311 322 10.1080/09540120500161983 16809108

[B21] Ross DA Changalucha J Obasi AI Todd J Plummer ML Cleophas-Mazige B Anemona A Everett D Weiss HA Mabey DC Grosskurth H Hayes RJ Biological and behavioural impact of an adolescent sexual health intervention in Tanzania: a community-randomized trial AIDS 2007 13 14 1943 1955 10.1097/QAD.0b013e3282ed3cf5 17721102

[B22] Cowan F Woelk G Chidiya S Ross DA Doyle A Hayes RJ Kaballa R Results and policy implications of two major adolescent sexual and reproductive health trials in Africa: The MEMA kwa Vijana Trial in Tanzania and the Regai Dzive Shiri Trial in Zimbabwe [satellite meeting] 15th International Conference on HIV and STIs in Africa. 30 November-7 December 2008; Dakar, Senegal

[B23] Plummer ML Wight D Obasi AI Wamoyi J Mshana G Todd J Mazige BC Makokha M Hayes RJ Ross DA A process evaluation of a school-based adolescent sexual health intervention in rural Tanzania: the MEMA kwa Vijana programme Health Educ Res 2007 13 4 500 512 10.1093/her/cyl103 17018767

[B24] Tanzanian Commission for HIV and AIDS The National Multisectoral Strategic Framework, 2004-2007. Dar es Salaam 2003

[B25] Simmons R Brown J Diaz M Facilitating large-scale transitions to quality of care: an idea whose time has come Stud Fam Plann 2002 13 1 61 75 10.1111/j.1728-4465.2002.00061.x 11974420

[B26] Reproductive Child Health sector of the Ministry of Health and Social Welfare The National Adolescent Health and Development Strategy 2004-2008. Dar es Salaam 2004

[B27] Cleophas B Obasi AIN Mshana G Wamoyi J Plummer ML Rwakatare M Ross DA Grosskurth H Gavyole A Evaluation of the youth-friendliness of reproductive health services in rural Tanzania using simulated patients [abstract no. WeOrD1277] 14th International AIDS Conference, 7-12 July 2002; Barcelona, Spain

[B28] Hallett TB White PJ Garnett GP Appropriate evaluation of HIV prevention interventions: from experiment to full-scale implementation Sex Transm Infect 2007 13 Suppl 1 i55 60 10.1136/sti.2006.023663 17215272

[B29] Nyonator FK Awoonor-Williams JK Phillips JF Jones TC Miller RA The Ghana community-based health planning and services initiative for scaling up service delivery innovation Health Policy Plan 2005 13 1 25 34 10.1093/heapol/czi003 15689427

[B30] Baraitser P Pearce V Walsh N Cooper R Brown K Holme J Smith L Boynton P Look who's taking notes in your clinic: mystery shoppers as evaluators in sexual health services Health Expectations 2007 13 1 54 62 10.1111/j.1369-7625.2007.00467.x PMC5060428 18275402

[B31] Grosskurth H Mosha F Todd J Mwijarubi E Klokke A Senkoro K Mayaud P Changalucha J Nicoll A ka-Gina G Newell J Grosskurth H Todd J Mayaud P Nicoll A Newell J Mabey D Hayes R Mosha F Senkoro K Changalucha J Klokke A Mwijarubi E ka-Gina G Mugeye K Impact of improved treatment of sexually transmitted diseases on HIV infection in rural Tanzania: randomised controlled trial Lancet 1995 13 8974 530 536 10.1016/S0140-6736(95)91380-7 7658778

